# Internet and Social Media Access Among Youth Experiencing Homelessness: Mixed-Methods Study

**DOI:** 10.2196/jmir.9306

**Published:** 2018-05-22

**Authors:** Lauren A Houdek VonHoltz, Rosemary Frasso, Jesse M Golinkoff, Alicia J Lozano, Alexandra Hanlon, Nadia Dowshen

**Affiliations:** ^1^ Children's Hospital of Philadelphia Philadelphia, PA United States; ^2^ College of Population Health Thomas Jefferson University Philadelphia, PA United States; ^3^ School of Nursing University of Pennsylvania Philadelphia, PA United States; ^4^ Craig-Dalsimer Division of Adolescent Medicine Department of Pediatrics Children's Hospital of Philadelphia Philadelphia, PA United States; ^5^ Department of Pediatrics Perelman School of Medicine University of Pennsylvania Philadelphia, PA United States

**Keywords:** adolescent, homeless youth, internet, social media, smartphone, health

## Abstract

**Background:**

Youth experiencing homelessness are at a risk for a variety of adverse outcomes. Given the widespread use of the internet and social media, these new technologies may be used to address their needs and for outreach purposes. However, little is known about how this group uses these resources.

**Objective:**

This study investigated how homeless adolescents use these technologies for general and health-related purposes, whether the scope of their use changes with housing status, and their interest in a website dedicated to youth experiencing homelessness.

**Methods:**

A convenience sample of youth aged 18 to 21 years was recruited from a youth-specific homeless shelter. All participants completed a 47-item survey, with 10 individuals completing a semistructured interview. Descriptive statistics, exact testing, logistic regression, and generalized estimating equation modeling was performed for quantitative data analysis. Interviews were transcribed verbatim, and NVivo 10 (QSR International) was employed to facilitate double coding and thematic analysis.

**Results:**

A total of 87 participants completed the survey with a mean age of 19.4 (SD 1.1) years. While experiencing homelessness, 56% (49/87) accessed the internet at least once a day, with 86% (75/87) accessing once a week. Access to a smartphone was associated with a 3.03 greater odds of accessing the internet and was the most frequently used device (66% of participants, 57/87). While experiencing homelessness, subjects reported a 68% decreased odds in internet access frequency (odds ratio [OR] 0.32, *P*<.001), 75% decreased odds in spending greater amounts of time on the internet (OR 0.25, *P*<.001), and an 87% decreased odds of social media use (OR 0.13, *P*=.01). Ten participants completed the semistructured interview. Several themes were identified, including (1) changes in internet behaviors while experiencing homelessness, (2) health status as a major concern and reason for Internet use, and (3) interest in a website dedicated to youth experiencing homelessness. While experiencing homelessness, participants indicated their behaviors were more goal-oriented and less focused on leisure or entertainment activities.

**Conclusions:**

While homeless youth experience changes in the frequency, amount of time, and specific uses of the internet and social media, study participants were able to access the internet regularly. The internet was used to search health-related topics. Given the importance of smartphones in accessing the internet, mobile-optimized websites may be an effective method for reaching this group.

## Introduction

An estimated 1.6 to 2 million youth in the United States experience homelessness yearly and often suffer adverse outcomes [1,2]. Youth experiencing homelessness (YEH) have a higher prevalence of mental health conditions such as depression, conduct disorders, posttraumatic stress disorder, and suicide attempts and ideation [3-7]. They are often the victims of physical and sexual abuse, engage in risky sexual behaviors, and are frequently diagnosed with sexually transmitted diseases including HIV [8,9]. Finally, YEH have higher rates of both substance use and dependence than their housed counterparts [10-12].

Numerous interventions have been designed to improve health and well-being of YEH including housing programs, substance use, mental health issues, and basic daily needs [13-16]. Although YEH often express interest in engaging in such programs and interventions, and often initiate participation, they meet challenges when trying to identify services, and once enrolled in programs, these youth have trouble staying engaged with service providers [17,18]. It is also difficult to establish a stable mode of contact as they often move frequently between different service organizations, shelters, and cities [2]. Additionally, transient living situations complicate service provision and make it difficult to engage youth in longitudinal research and evaluation of interventions in this population [19].

Given the ubiquity of internet and social media use among adolescents in the United States, mHealth technologies represent a unique opportunity for interventions to improve health outcomes among YEH [20-23]. The utilization of these technologies as a novel approach to intervention demonstrates promise as preliminary work in the field suggests that YEH are able to access the internet with relative frequency [24,25]. A recent study indicated that approximately 80% of YEH used the internet at least once per week, with nearly 25% using the internet for at least an hour daily [25]. With regard to health-seeking behaviors, recent reports demonstrated that YEH use the internet to find information regarding HIV and other sexually transmitted infections (STIs) as well as looking for STI testing services [25].

Although these studies provide support for the use of the internet and various forms of social media as a viable method of connecting with YEH, several questions remain. More information is needed to determine if there are differences in youths’ rates of access to the internet and social media before a homeless episode as compared with when they are in the midst of an episode. It is possible that those who face homelessness may have already been disenfranchised regarding access to these technologies. Additionally, understanding differential access methods between the 2 time periods (prehomelessness and during homelessness) may provide insight on how to best reach these youth during episodes of homelessness as well as help determine preferred contact and outreach methods. Furthermore, understanding what information this group is seeking and how they seek it can inform interventions to improve health outcomes. Therefore, this study utilized a mixed-methods approach to examine rates of internet access, duration of internet use, devices used to access the internet, and characterize specific internet behaviors among a sample of youth before and during an episode of homelessness. We hypothesized that participants would have less access and less frequent use of the aforementioned technologies while experiencing homelessness. We also sought to identify health-related behaviors YEH engage in on the internet. Finally, using qualitative semistructured interviews, we explored how technology could be utilized to reach YEH to accurately and efficiently provide resources appropriate for YEH.

## Methods

### Setting and Sample

We engaged participants living at a nonprofit, youth homeless shelter in Philadelphia that annually serves approximately 350 youth aged 18 to 21 years. The facility provides services such as education and vocational advisement, an on-site medical clinic, and case management to the youth residing in the shelter. Study participants were 18-21 years old, housed at the shelter at the time of the study, and English speaking. Individuals were excluded if they were visibly intoxicated or unable to participate due to medical or psychosocial distress at the time of the interview. Only 1 subject was excluded for intoxication. Enrollment occurred between July 14 and September 12, 2014, Monday-Friday, 9 AM-5 PM. The team employed a convenience sampling strategy, and residents of the shelter were invited to participate in the study at on-site meal-time gatherings. All surveys and interviews were conducted in a private room in the shelter. This study was approved by the University of Pennsylvania Institutional Review Board.

### Data Collection

All participants were considered homeless during this study by the investigators. Throughout the survey and interview, participants were asked to compare their internet, social media, and mobile health app usage during this period of homelessness to a time they did not consider themselves homeless and had stable housing.

This study employed a complementary qualitative and quantitative data collection approach, wherein quantitative and qualitative data were collected over the same period [26].

#### Quantitative Data Collection

A 47-item survey was administered to all study participants. Questions and answer choices were read aloud to minimize literacy concerns. Study data were collected and managed using a secure electronic database [27]. The survey took 10-15 min to complete and included topics such as age, gender, race, and sexual orientation. Internet and social media use and Web-based health-seeking behaviors during and before the participant’s current homeless episode were assessed. Respondents were asked to indicate their interest in a website or app that focused on the needs of YEH (see Multimedia Appendix 1 for full survey). Participants were given a US $10 gift card for participation and only completed a single survey.

#### Qualitative Data Collection

Following quantitative data collection, approximately every fifth participant (n=10) was invited to complete a semistructured, approximately 45-min interview in a private office at the shelter with a member of the research team (LV). We employed a constant comparison approach, that is, we reviewed and analyzed the transcripts as data were collected, to determine if new information was emerging [26]. After 10 participants were interviewed, analysis revealed that theme saturation had been reached, and no further participants were recruited. The most common reason for declining to participate was the length of the interview. The interview guide was informed by the literature and expert consultation [25,28,29]. All interviews were audio recorded and began with questions regarding the participant’s current and historical access to the internet. Questions were designed to explore frequency of internet use during this episode of homelessness, specific access methods (ie, library computer, smartphone), time spent using the internet, and compare their current experience to that when they were housed. Respondents were also asked to comment on how and for what purposes they used the internet and social media, and whether they felt there were any differences between their current usage patterns and their patterns of use before this shelter stay. Other questions addressed health concerns and behaviors, the frequency of health-related thoughts, and health-related internet searches. Participants were asked whether they would use an app or website with content specifically designed to support YEH. They were asked to suggest content and resources, comment on format preference (app or website), and share any other suggestions that would make this type of resource useful to them. Participants were given a US $20 gift card for their participation in the interview.

### Analyses

#### Survey Analysis

Descriptive statistics were used to characterize the sample. Means and standard deviations were used to describe continuous variables, and frequencies and percentages were used to describe categorical variables. Fisher exact tests were used to examine differences in demographics between participants who completed the survey and follow-up interview versus those who only completed the survey; two-sample *t* tests were used to compare continuous variables across the 2 groups. Generalized estimating equation (GEE) regression was used to model categorical internet use variables (internet access frequency, time spent using the internet, use of social media, access to a smartphone) at 2 time points, specifically before and while experiencing homelessness. Although surveys were conducted only once, study participants were asked to answer the same questions about access before and while experiencing homelessness. GEE methodology allows for modeling of the marginal distribution of each categorical outcome variable as a function of homelessness, while adjusting for covariates, and accounts for the likely correlations of the repeated outcome measures for each participant. Finally, 2 separate logistic regression models were used to examine the association between having access to a smartphone on the odds of internet access at least once a day in participants before and while experiencing homelessness. All models were adjusted by age, male gender, and sexual orientation. Statistical significance was taken at *P*<.05. All statistical analyses were conducted using SAS Version 9.4 (SAS Institute Inc, Cary, NC).

#### Interview Analysis

Interviews were transcribed verbatim and entered in NVivo 10 software (QSR International, Melbourne, Australia) to facilitate thematic coding and systematic analysis [30]. A coding dictionary was developed inductively following careful reading of a set of representative transcripts by 3 members of the study team (LV, JG, and RF) [31]. See Multimedia Appendix 2 for coding tree and codebook. Specific codes identified in this study include internet access, cost, employment, housing, commercial resources, social media, emotions related to access, mobile phone app s, health activities, and suggestions for a YEH-specific website. Each subsequent interview was double-coded by 2 members of the study team (LV and JG). Evaluation of intercoder reliability was supported by NVivo 10 software employing the kappa coefficient. The software compares agreement and disagreement between coders in the assignment of specific codes to segments of the interview transcript. Complete agreement in coding correlates with a mean kappa of 1; near perfect agreement, a mean kappa of .81-.99; substantial agreement, a mean kappa of .61-.80; and moderate agreement, a mean kappa of .41-.60. Analysis of intercoder reliability for this study revealed substantial to almost perfect agreement (mean κ=.94; range, .76-1.00). This result was supported by percentage of agreement analysis, which yielded a mean of 99% (range, 92-100%) agreement of all codes examined in this study. After coding was complete, the study team organized the codes into thematic categories described later in the paper.

## Results

### Quantitative Results

#### Participant Demographics

Participant demographics for the sample have been summarized in Table 1. A total of 87 youth completed the survey. Respondents were 18-21 years old, with a mean age of 19.4 years (SD 1.1). The sample consisted of 60% (52/87) males, with 75% (65/87) identifying as African American or black and 14% (12/87) identifying as a sexual minority. No statistically significant differences in demographics were observed between participants who completed the survey and a follow-up interview and those who only completed the survey (Table 1).

#### Internet Use, Social Media Use, and Health-Seeking Behavior While in Shelter

While experiencing homelessness, 86% (75/87) participants were able to access the internet at least once a week and 56% (49/87) of youth were able to access the internet at least once a day. The majority of youth surveyed spent less than an hour using the internet each day, with smartphones (66%, 57/87) and public computers (59%, 51/87) being the most commonly used devices for access. Participants also used the internet for job searching (86%, 75/87), checking email (83%, 72/87), searching for housing (60%, 52/87), and browsing school websites (51%, 44/87). The majority of youth utilized social media (85%, 74/87). Significant differences in internet access frequency (*P*<.001), amount of time spent using the internet each day (*P*<.001), access to a smartphone (*P*<.001), and use of social media (*P*=.01) were observed before and while experiencing homelessness (Table 2).

#### Comparison of Internet Use Characteristics Before and During Current Shelter Stay

Adjusted GEE model results demonstrated that current homelessness was significantly associated with a lower odds of regular internet access frequency, more time spent using the internet, use of social media, and access to a smartphone (see Multimedia Appendix 3). Specifically, participants reported a 68% decreased odds in internet access frequency of at least once a day during their current episode of homelessness as compared with before experiencing homelessness, adjusting for age, male gender, and sexual orientation (odds ratio [OR] .32, 95% CI 0.18-0.57, *P*<.001). Compared with before experiencing homelessness, participants reported a 75% decreased odds in greater amounts of time spent using the internet during their current episode of homelessness, adjusting for age, male gender, and sexual orientation (OR 0.25, 95% CI 0.16-0.41, *P*<.001). Participants reported an 87% decreased odds in use of social media while experiencing homelessness compared with before experiencing homelessness, adjusting for age, male gender, and sexual orientation (OR 0.13, 95% CI 0.03-0.55, *P*=.01). Finally, participants reported a 67% decreased odds in access to a smartphone during their current episode of homelessness compared with before experiencing homelessness, adjusting for age, male gender, and sexual orientation (OR 0.33, 95% CI 0.18-0.60, *P<*.001).

Furthermore, adjusted logistic regression models demonstrated that although smartphone access was significantly associated with greater odds of regular internet access while experiencing homelessness (*P*=.02), it had no influence on a participant’s ability to use the internet before experiencing homelessness (data not shown; *P*=.38). Specifically, the odds of internet access at least once a day among participants with access to a smartphone while experiencing homelessness was 3.03 times that of someone without a smartphone, adjusting for age, gender, and sexual orientation (OR 3.03, 95% CI 1.17-7.84).

**Table 1:** Participant demographics: youth living in a young adult homeless shelter in Philadelphia.

Characteristic		All participants (N=87)	Survey and follow-up interview participants (n=10)	Survey-only participants (n=77)	*P* value^a^
**Age in years, mean (SD)**		19.4 (1.1)	19.3 (1.2)	19.4 (1.1)	.71
**Gender, n (%)**					.12
	Male	52 (60)	5 (50)	47 (61)	
	Female	34 (39)	4 (40)	30 (39)	
	Transgender	1 (1)	1 (10)	0 (0)	
**Race, n (%)**					
	African American or black	65 (75)	7 (70)	58 (75)	.71
	White	10 (12)	1 (10)	9 (12)	.99
	American Indian or Alaska Native	5 (6)	0 (0)	5 (7)	.99
	Other	19 (22)	3 (30)	16 (21)	.68
**Hispanic, n (%)**					.45
	Yes	23 (26)	4 (40)	19 (25)	
	No	64 (74)	6 (60)	58 (75)	
**Sexual orientation, n (%)**					.12
	Heterosexual	74 (85)	8 (80)	66 (86)	
	Gay	3 (3)	1 (10)	2 (3)	
	Lesbian	1 (1)	1 (10)	0 (0)	
	Bisexual	8 (9)	0 (0)	8 (10)	
	Prefer not to answer	1 (1)	0 (0)	1 (1)	

^a^*P* values based on Fisher exact tests for categorical variables, and two-sample *t* tests for continuous variables.

**Table 2:** Internet use characteristics of youth living in a Philadelphia youth homeless shelter comparing before experiencing homelessness to while experiencing homelessness.

Characteristic		Before experiencing homelessness (N=87)	While experiencing homelessness (N=87)	*P* value^a^
**Internet access frequency, n (%)**				<.001
	≥once/day	69 (79)	49 (56)	
	<once/day	18 (21)	38 (44)	
**Amount of time spent using the internet daily, n (%)**				<.001
	<20 min	8 (9)	23 (26)	
	20 min-1 h	20 (23)	32 (37)	
	1-2 h	10 (12)	11 (13)	
	>2 h	49 (56)	21 (24)	
Access to smartphone, n (%)		74 (85)	57 (66)	<.001
Job searching, n (%)		81 (93)	75 (86)	.11
Use of social media (Facebook, Twitter, and Instagram), n (%)		85 (98)	74 (85)	.01
Use of email, n (%)		79 (91)	72 (83)	.07
Housing searches, n (%)		55 (63)	52 (60)	.56
School website searches, n (%)		55 (63)	44 (51)	.06

^a^*P* values based on unadjusted GEE regression models to account for the likely correlations of repeated measures for each participant.

#### Interest in a Website or App Focused on Youth Experiencing Homelessness

Survey respondents were asked if they would use a website or app if one were designed to focus specifically on issues facing YEH. A total of 91% (79/87) of the participants indicated that they would use a website or app specifically designed to support YEH.

### Qualitative Results

We conducted semistructured interviews with 10 study participants that were representative of the larger sample (Table 1). Coding of interview transcripts revealed several important themes (which capture and organize constructs and experiences). The study team then met and organized the codes into the following thematic categories: (1) impact of homelessness on internet activities, (2) focus on health status, and (3) interest in a youth homelessness website. The following paragraphs summarize the findings for each of these categories followed by illustrative quotes.

#### Impact of Homelessness on Internet Activities

The majority of interviewed youth noted that there were striking differences in how they currently use the internet and social media compared with before experiencing homelessness. Consistent with survey findings, participants reported less frequent access of the internet and less time spent using the internet. However, the interviews revealed that the decrease in internet activity was nuanced. Specifically, participants experienced more substantial decreases in entertainment or leisure activities. internet activities became more goal oriented with participants using most of their time on the internet to look for basic needs such as housing, food, and employment. Finally, the youth indicated that time restrictions on public computers impacted their internet activities by making them more purpose driven:

It’s not so much for leisure or entertainment anymore. It’s like I'm going to there with a purpose now. I'm really looking for, or waiting for certain information to help me get where it is I'm trying to go.

Male, 21 years

Several participants spent time discussing how their perception of their housing security affected how they used the internet. When they considered their living situation to be more tenuous and unstable, the internet was regarded as an essential resource. Participants indicated they would use the internet to contact people who might help them with housing/or to find food resources. When their living situation became more secure, the internet could then be used more for entertainment purposes. Conversely, one participant indicated that now that she had found stable housing through the shelter, she rarely used the internet. For this youth, the internet was exclusively used as a resource during times of crisis:

It’s [the internet] really more important when you're struggling, because you need it. It’s like you need it, because you need to keep in contact and you need to make sure you write. You need it for a lot, than when you're not struggling [with housing].

Female, 19 years

#### Focus on Health Status

Participants indicated they thought about their health frequently, with most (9/10) indicating they thought about their health daily. For many participants, health was a source of preoccupation and worry. The participants discussed being stressed about developing various acute illnesses or other diseases, worrying about current health conditions such as asthma and HIV, focusing on their physical fitness, and finding ways to stay healthy.

Participants used the internet as a resource to find information about acute illnesses, child development, alternative medicine and remedies, cancer, exercise, nutrition and calories, provider and hospital contact information, and STIs. Although the youth were able to easily find information on the internet about these various topics, many reported having trouble interpreting the results of their searches. Often, they found the information was presented using complicated terminology that was meant for health care professionals. Some described working around this limitation by looking up synonyms for the words they did not understand. Others expressed frustration at the sheer volume of information that was available and the difficulty they experienced when trying sorting through the results of an internet search on a topic:

Like I'll be thinking like if I do certain things, how it’s going to affect me on the inside, or like I'll be wondering if I'm sick or if I'm about to take a pill or something, I'll hurry up and Google it, to see what it’s going to do to my insides. I do it all day.

Male, 20 years

The youth were asked about using social media as a means to connect with health care providers or clinics. The group had mixed views; some opposed the idea, whereas others thought it would be a convenient way to reach their physicians. Email and phone conversations were mentioned as preferred methods of contact:

I don’t know. I don’t feel like it’s professional for me to talk to a doctor on Facebook. It’s not really my way of looking at things. I feel like they should be a business or somebody I should call, not on Facebook.

Male, 18 years

#### Interest in a Youth Homelessness Website

All participants expressed interest in the development of a website or app that would aggregate resources and address topics to help YEH. They also indicated they would use this resource and recommend it to friends. The youth suggested many topics, including information about reliable housing, job opportunities, and women’s and men’s health issues. They also recommended that youth living at the shelter be involved in website development. A full list of potential website topics that were suggested by participants can be found in Textbox 1.

Participants also suggested using social media, YouTube, and word of mouth as ways to ensure the website or app reached the people who would be most in need of the resources:

I think it’s [the website] cool, because, honestly, we can use all the help we can get.

Female, 18 years

**Suggested topics for youth homelessness website obtained from youth who completed an interview while living in a Philadelphia youth homeless shelter.**

- Housing
  - Shelters and emergency housing
  - Legitimate housing and reliable landlords

- Food pantries and kitchens

- Classifieds and job opportunities

- Health
  - Women’s and men’s health sections
  - Clinic information, planned parenthood locations
  - Sexually transmitted infection information
  - Substance use information

- General Equivalency Diploma and education resources

- Important phone numbers

- Services

- Serve as a Web-based repository for important information (eg, immunization records, school transcripts)

## Discussion

### Principal Findings

The results of this study contribute to a small volume but growing body of literature that investigate how to best reach and engage YEH. Consistent with previous research, this study indicated that YEH are able to access the internet and social media with relative frequency, though less often than when they were housed [32]. Smartphones and public computers were heavily utilized for internet access during episodes of homelessness and shelter stays. Smartphone ownership had a strong impact on internet activities, as youth in the shelter with access to a smartphone were able to use the internet and social media more consistently than their peers who did not have smartphone access.

A significant body of research in this area has emerged from the work of Rice and colleagues in Los Angeles, CA [25,28,29]. Our study provides a perspective from a very different geographic location and captures the experience of a predominantly minority demographic. Despite the differences in study populations, several similar themes emerged. YEH are utilizing internet access for a multitude of activities including connecting with family and friends on social media, entertainment, job searching, and health-related searches. A trend among this study’s population of shelter-housed youth was the use of the internet in a more goal-directed fashion. The internet was more frequently used as a tool to seek out resources related to housing and employment rather than as a source of entertainment during periods of housing insecurity. This highlights how these various technologies are vital tools for homeless youth as they work to obtain a more secure financial footing during times of housing strife. These goal-directed behaviors deserve recognition by policy makers when they are considering legislation that affects public internet access and the importance of reliable and regular internet access among this vulnerable group of young people.

The results of this study also highlight the importance of personal health among study participants. However, many homeless youth lack access to the health care system due to a lack of health insurance. Additionally, many do not utilize available services because they are addressing more pressing needs such as finding food and housing or due to perceived and actual barriers to accessing medical care [33]. As many youth are already turning to the internet for health concerns, there exists an opportunity to create a resource for homeless youth. Previous researchers created a Web-based personal health information system for YEH that allowed participating individuals a safe space to store confidential health information and direct access to public health nurses [34]. The interface was well received by study participants and created a safe environment for addressing many of the complex health needs the adolescents face. This study, like ours, identified an interest in reliable resources regarding health and a need among YEH. In our study, both the survey and qualitative data revealed the majority of participants used the internet as a primary source of information for health-related matters, and that health was very important to those interviewed. Future work in this area can focus on dependable information about common adolescent health issues and about available local resources in a central location.

In addition to a resource that focuses on the health needs of YEH, this study identifies the need and interest in an internet-based platform that addresses the multitude of challenges faced by homeless youth. The results from the qualitative portion of the study further elucidated that limited phone data plans and computer time limits at public libraries had a strong influence on their internet behaviors. As the prevalence of smartphone use is high in this population, any internet outreach efforts must be specifically designed to be compatible with mobile devices. Websites that do not work well on mobile devices may discourage use and reduce their desired outreach effect.

This research stresses the importance of digital inclusion, especially with regard to this vulnerable population. Digital inclusion refers to the ability of groups and individuals to gain access to the internet, identify appropriate material and resources, as well as have opportunities for training to obtain the necessary skills to effectively use available material [35]. This study draws attention to significant opportunities to enhance digital inclusion among YEH. Specific opportunities include website content that is relevant to the acute needs of YEH, creating websites that reflect access patterns, and increasing access points to expand the reach of resources and services. Public policy efforts, libraries, and research have sought to understand and increase digital inclusion; however, there remains significant opportunity for improvement among YEH [36].

### Limitations

There are several limitations to this exploratory study. Given that participants currently in shelter were asked to recall behavior before experiencing homelessness, responses may have been subject to recall bias. Additionally, because participants were all recruited from the same youth shelter in one city, results may have limited transferability and generalizability. Our sample size was relatively small with only 87 subjects, which led to wide confidence intervals throughout analysis. Future work can seek to examine internet use among larger samples and more diverse demographics of YEH to better establish specific types of interventions that would be the most useful. Despite these sample size limitations, among similar studies recruiting YEH, this study has a comparable sample size. Additionally, it is among one of the first and largest mixed methods studies to describe access to the internet and social media among a sample of YEH in a major Northeastern US city.

### Conclusions

Although the overall time spent using the internet and utilizing social media sites was decreased compared with the time in which they were not struggling with homelessness, the young people in our sample were able to use these resources with relative frequency. Smartphones and public libraries were instrumental in providing access. Moving forward, mobile device friendly, adolescent-centered Web platforms, may serve as an important resource to providing services to and connecting with young people struggling with homelessness.

## Appendix

Multimedia Appendix 1 Internet use survey.

Multimedia Appendix 2 Internet and youth homelessness codebook and coding tree.

Multimedia Appendix 3 GEE model results.

## Abbreviations

GEE: generalized estimating equation
OR: odds ratio
STI: sexually transmitted infection
YEH: youth experiencing homelessness
