# Supplementary material for: Internet and Social Media Access Among Youth Experiencing Homelessness: Mixed-Methods Study
Source: J Med Internet Res. 2018 May 22;20(5):e184. doi: 10.2196/jmir.9306 (PMC5989062; doi:10.2196/jmir.9306)
Supplement: Multimedia Appendix 1 [file jmir_v20i5e184_app1.pdf]

Multimedia Appendix 1: Internet Use Survey

Introduction: We are giving out this survey to better understand how young adults and teens in your living situation use the internet, what websites are visited, and whether any of the activities are related to health. This survey will ask you some basic questions about yourself and then go into questions about how you use the internet at different time periods in your life. For each question, I will read the whole question and then the answer choices. You do not have to participate in this survey to continue to receive services where you are living. This survey is completely voluntary. You may stop at any time and refuse to answer any questions that make you uncomfortable. Do you have any questions?

1. How old are you?

______

2. What is your gender?
   - o Male
   - o Female
   - o Transgender
   - o Other
   - o Prefer not to say/Don't want to say

3. Are you Hispanic or Latino?
   - o Yes
   - o No
   - o Not sure

4. What is your race?
   - o American Indian or Alaska Native
   - o Asian
   - o Black or African American
   - o Native Hawaiian or Other Pacific Islander
   - o Other Race
   - o White

5. What best describes your sexuality?
   - o Straight
   - o Gay
   - o Lesbian
   - o Bisexual
   - o Not sure
   - o Other
   - o Prefer not to say/Don't want to say

6. Right before living here (**Insert shelter site**), what best describes your living situation?
   - o Living with parent or guardian in our home/apartment
   - o Living with brothers or sisters, but no parents
   - o Living with friends
   - o Living in another shelter
   - o Living on the streets or an abandoned building
   - o Living in a car

- o At a hospital
- o Living in a jail/prison
- o Living with a spouse or partner

7. How long were you living in this situation before you came here?

8. How long have you been living here?

—

9. Do you consider yourself homeless?
- o Yes
- o No

10. If yes, how long have you been homeless?

—

<u>I am now going to ask you questions about your use of the internet. Using the internet means using an internet connection such as cable line, WIFI or a 3G/4G network to do things like check email, go on websites, check Facebook, Instagram and Twitter. It also means that you are doing this on a device like a smartphone, computer, or tablet. If you want to know whether something counts as using the internet, please ask.</u>

11. After hearing the above definition of what it means to use the internet, have you used the internet?
- o Yes
- o No

<u>The next few questions will ask about how you used the internet before becoming homeless</u>

12. Before becoming homeless, which choice best describes how often you were able to use the internet?
- o Many times a day
- o At least once a day
- o 3-5 times a week
- o 1-2 times a week
- o Every 1 to 2 weeks
- o Every 3 to 4 weeks
- o It was more than a month between each time I used the internet

13. Before becoming homeless, what choice describes the amount of time you spent on the internet in a typical day?
- o Less than 20 minutes
- o 20 minutes to 1 hour
- o 1 to 2 hours
- o More than 2 hours

14. Before becoming homeless, what did you use to get onto the internet? Check all that apply.
- o Smartphone
- o Your own computer
- o Public computer (such as one at a library)

- Computer at school
- Computer at work
- Tablet (such as iPad, Microsoft Surface)
- Regular cell phone
- Other:

15. Before becoming homeless, what did you use **most often** to get onto the internet? Check only one answer.
- Smartphone
- Your own computer
- Public computer (such as one at a library)
- Computer at School
- Computer at Work
- Computer at School
- Computer at Work
- Tablet (such as an iPad, Microsoft Surface)
- Regular cell phone
- Other:

16. If you were using a smartphone to go online, whose phone was it?
- My phone
- My mother's
- My father's
- My brother or sister
- A friend
- Other:

17. Before becoming homeless, did you visit any of these websites? Check all that apply.
- Facebook
- Instagram
- Twitter
- Yelp
- Foursquare

18. Before becoming homeless, did you do any of these things while using the internet? Check all that apply.
- Check email
- Look for jobs
- Look at school websites (such as colleges or training programs)
- Look for a place to live (such as an apartment or housing program)
- Look up information about my health
- Other:

The next few questions will ask about how you use the internet since becoming homeless

19. Since becoming homeless, which choice best describes how often you were able to use the internet?
- Many times a day
- At least once a day
- 3-5 times a week

- 1-2 times a week
- Every 1 to 2 weeks
- Every 3 to 4 weeks
- It was more than a month between each time I used the internet

20. Since becoming homeless, what choice describes the amount of time you spend on the internet in a typical day?
    - Less than 20 minutes
    - 20 minutes to 1 hour
    - 1 to 2 hours
    - More than 2 hours

21. Since becoming homeless, what do you use to get onto the internet? Mark all that apply
    - Smartphone
    - Your own computer
    - Public computer (such as one at a library)
    - Computer at school
    - Computer at work
    - Tablet (such as iPad, Microsoft Surface)
    - Regular cell phone
    - Other:

22. Since becoming homeless, what do you use **most often** to get onto the internet? Check only one answer.
    - Smartphone
    - Your own computer
    - Public computer (such as one at a library)
    - Computer at school
    - Computer at work
    - Tablet (such as an iPad, Microsoft Surface)
    - Regular cell phone
    - Other:

23. If you are using a smartphone to go online, whose phone is it?
    - My phone
    - My mother’s
    - My father’s
    - My brother or sister
    - A friend
    - Other:

24. Since becoming homeless, have you visited any of these websites? Check all that apply.
    - Facebook
    - Instagram
    - Twitter
    - Yelp
    - Foursquare

25. Since becoming homeless, do you do any of these things while using the internet? Check all that apply.

- o Check email
- o Look for jobs
- o Look at school websites (such as colleges or training programs)
- o Look for a place to live (such as an apartment or housing program)
- o Look up information about my health
- o Other:

26. Since becoming homeless, what websites do you visit **most often**? Check only one answer choice.
- o Email
- o Social Media (ex. Instagram, Facebook, Twitter, Tumblr, SnapChat)
- o Job websites
- o Websites with housing information
- o News Websites
- o School websites (ex. college websites, high school, middle school)
- o Health websites (ex. WebMD, doctor's office websites)
- o Other:

The next few questions are about the last time you were on the internet

27. The last time you were on the internet, where were you?
- o Library
- o School
- o Family home
- o Friend's house
- o Where you live right now
- o Cafe or Coffee House
- o Work
- o Other:

28. The last time you were on the internet, what were you using?
- o Smartphone
- o Your personal computer
- o A public computer (such as one at a library)
- o Computer at school
- o Computer at work
- o Tablet (such as an iPad, Microsoft Surface)
- o Regular Cell phone
- o Other:

29. The last time you were on the internet, how long did you stay on?
- o Less than 20 minutes
- o 20 minutes to 1 hour
- o 1 to 2 hours
- o More than 2 hours

30. The last time you were on the internet, were you able to stay on as long as you wanted?
- o Yes
- o No

31. If you were not able to stay on as long as you wanted, why?
- Internet time limits on the computer
- Ran out of time on data plan
- Interrupted (*For example by a phone call, a family member, needed to leave to go to an appointment)*
- Other:

32. The last time you were on the internet, what websites did you visit?

33. The last time you were on the internet, did you visit any of these websites?
- Email
- Facebook
- Instagram
- Twitter
- Yelp
- Foresquare
- News websites
- School websites (such as colleges, GED programs, or training programs)
- Job search
- Websites with housing information (apartment websites, Craigslist)
- Health information websites
- Other:

<u>The next few questions will ask about ways you may or may not look for information on the internet about your health</u>

34. Do you use the internet or social media to find any information about your general health? Examples of social media include Facebook, Twitter, Instagram
- Yes
- No

35. If yes, what do you use?
Please list:

36. Do you use the internet or social media to find a doctor?
- Yes
- No

37. If yes, what do you use?
Please list:

38. Do you use the internet or social media to find any information about your sexual health? *This can include, but isn't limited to, things like sexually transmitted disease testing, HIV testing, pregnancy testing, and prenatal or pregnancy care.*
- Yes
- No

39. If yes, what do you use?

40. Do you use the internet or social media to find any information about substance use?

This can include, but isn't limited to, information about rehab programs, help with quitting drug or tobacco, finding support groups, or needle programs.

- Yes
- No

41. If yes, what do you use?

42. Do you own a smartphone?

- Yes
- No

43. If yes, please list some of the apps you have:

44. Do you have any apps that you use to help you stay healthy?

- Yes
- No

45. If yes, what are they?

46. Do you have any of these apps?

- Calorie Counter App
- Exercise Apps
- Asthma Apps
- Apps for your period (only asked to females)
- Pregnancy Apps
- Apps to help with sleep
- Apps to quit smoking
- Apps that have your medical records

47. Would you use a website or app that focused on issues homeless young adults face?
Such a website or app may include a list of resources such as important phone numbers, available services, advice from others, and lists of potential jobs.

- Yes
- No
- Maybe
