# Supplementary material for: Internet and Social Media Access Among Youth Experiencing Homelessness: Mixed-Methods Study
Source: J Med Internet Res. 2018 May 22;20(5):e184. doi: 10.2196/jmir.9306 (PMC5989062; doi:10.2196/jmir.9306)
Supplement: Multimedia Appendix 2 [file jmir_v20i5e184_app2.pdf]

# Multimedia Appendix 2: Internet and Youth Homelessness Codebook and Coding Tree

| Node | Definition |
|---|---|
| Access method | ***Use this node when subject identified what device he/she used to get online. Use this node if there is a specific mention of whether they used Wifi vs. data plan or a library computer***<br><br>Ex. I: What do you typically use to get on the internet?<br>R: My phone<br><br>***Add the negative – what they aren't using to access the internet***<br><br>Ex. I've noticed that now that I'm homeless, I don't post any statuses. That's changed for me. Not that I don't have a phone either, I don't post any statuses. |
| Apps | ***Use this to include both health and non-health related apps used by the subject. Also include ideas for future apps.***<br><br>I: Do you have any apps on your phone that you use to help you stay healthy?<br>R: My UV ray thingy. I have a UV ray app that tells me the best times to go outside and not get skin damage basically. |
| Commercial Resources | ***Use this node when subject comments on methods of internet access from commercial locations.***<br><br>This includes McDonald's, Laundromat, etc<br><br>Ex. I: What are some places you go to find Wifi?<br>R: McDonald's, the library, Dunkin Donuts, any coffee shop, the Laundromat down the street. |
| Convenience/lack of convenience in accessing the internet. | ***Use this to reference when it is easy or convenient (or not easy and convenient) to access the internet and other technology. This can include the ease of getting a library card and library internet use.***<br>***Include things that are making it difficult to get online***<br><br>***Do not include ease/lack of ease of accessing certain types of websites and internet activities***<br><br>I: Is there anything that makes it easy for you to get on the internet?<br>R: The accessibility of my phone. It's right there, modern technology, easiness. |
| Cost of access | ***Use this node when the subject discusses financial challenges of accessing the internet, cost of a phone and phone plan***<br><br>Ex. R: So money is a problem, because if I had money, I could buy myself a hot spot, or I could-yeah, I could do those |

| | |
|---|---|
| | things, but I can't. |
| Employment | ***Use this node when the subject discusses a job they have, job searching, importance of a job, benefits associated with a position etc.***<br><br>***Include descriptions of the military***<br><br>***Things you can do to be eligible for a job.***<br>I: What are the most important things for you to do when you're on the internet?<br>R: Job applications, applications for like the welfare office and stuff like that, so like assistance, like medical assistance and food stamps and all those things. |
| Feelings and emotions related to access and use of the internet | ***Use this node when subject discusses any emotion (negative or positive) related to being able to get online, not being able to get online, losing a method of access, etc. This can also involve reactions to losing a phone that was a method of internet access.***<br><br>***Also include additional feelings not restricted to accessing the internet.***<br><br>***Make sure the respondent is clearly expressing an emotion – ex. "I do this for fun, this makes me angry, I love the internet".***<br><br>***Patience and mindfulness are also feelings***<br>Ex. I: And has there ever been a time where you didn't have your phone?<br>R: Yes, and I was depressed.<br><br>I: Do you ask them on their wall or do you send them a message?<br>R: I send them a message. I really don't like writing on people's walls. It makes me irritated, because I don't like when people write on my wall because it's supposed to be about me. |
| General assistance | ***Use this node when subject comments on freely available community services both non-profit and government based.***<br>***Can also add characteristics of the shelter they are living in: what services the shelter provides, what you can/can't do at the shelter.***<br><br>***Do not include hypotheticals – discussions of potential community resources that might be helpful.***<br>This includes all types of shelters (both city and independently funded), all types of food assistance (both soup kitchens and federal food stamps), the library, job service centers, hygiene services, transportation assistance, etc.<br>Housing: Shelters (additional housing information will go in the housing section) |

| | |
|---|---|
| | Hygiene: public showers, cleaning product distribution<br>Food: soup kitchens, pantries,<br><br>Ex. R: But then now times, I use the library computer of CareerLink's. *Note this example would also be coded as access |
| Goals/Aspirations for the future | ***Use this when subjects discusses future plan, career goals, goals post homelessness, etc.***<br><br>Travel goals<br><br>Ex. R: I want my own house and my own kids and my own care and stuff like that, and medical insurance and everything else in the world. |
| Good Quotes | Flag good quotes |
| Health | ***Use this to record what activities a subject does to maintain his/her health, what health related websites they visit, impact of these activities on behavior, mental health, health education, connecting with a provider (this includes hypothetical connections with a provider)***<br><br>***Can include descriptions of how health apps help you manage your health.***<br><br>I: Do you use any other websites or resources to help you when you're thinking about your health?<br>R: I like Web MD. I like health websites, like the health news, like in the newspapers and Google and stuff like that. |
| Health literacy/literacy demands | ***Specifically one's ability to process information related to health – could be written or oral communication. Could refer to online or offline communication.***<br><br>Ex. I: Do you feel like the information you're finding is directed at your level, or is it in like these strange terms.<br>R: Strange terms…I wish you would just say something normal like, you have a cold, not you have whatever a cold is in the doctor term or scientific term. That's ridiculous. I don't know what that is. |
| Housing | ***Use this node when the subject discusses looking for housing, needing housing, methods of obtaining housing, types of available housing, etc.***<br><br>Get thrown out<br><br>Ex. R: And I look up a lot of like housing things… |
| Independence/Autonomy | ***Use this node when the subject discusses the desire for independence, ways to obtain/maintain independence/autonomy, managing his/her own affairs, not needing resources from others.***<br>***Independence in housing and finances (ie having a job) is also included.*** |

| | |
|---|---|
| | Ex. R: Because I can't live without my phone, because I don't like to rely on like the system basically. The staff here, if you – it's just really hard to get what you need to get done. |
| Pre-homelessness vs. Homelessness | ***Use this node when subject compares pre-homelessness activities to activities they are engaging in now that they are homeless.***<br><br>This includes internet related behaviors, health related behaviors, interactions with others, etc.<br><br>Ex. I: …is there any difference in what you use to get online?<br>R: Yeah, because usually I would just get on my phone, but now I get on my phone and computers and stuff like that at the library.<br>I: Did you ever use computers before you started struggling with housing?<br>R: I used computers, just not as much. |
| Privacy/Security | ***Use this when the subject discusses concerns regarding protection of or threats to their digital or device privacy/security.***<br><br>***Ex.***<br>I: Do you have any worries about privacy or anything with that kind of a system?<br>R: Not with DM'ing. DM'ing is different, because it's direct message to you and only you. But then the things that we post on your wall, everybody will see. |
| Social commentary | ***Use this when the subject makes sweeping statements about society or a group of people.***<br><br>Ex. That's what everyone wants…<br>R: Nobody goes on Facebook anymore. And if they do, it's because that have like a bajillion friends on Facebook, and it's because they want to be Facebook famous. |
| Social Media | ***Use this node to document types of social media websites subjects visit, as well as activities engaged in on these websites.***<br><br>***Include when people talk about keeping in touch with friends/family on social media sites***<br><br>Examples of social media sites include: Facebook, Twitter, Instagram, Kik, MocoSpace, SnapChat |
| Suggestions for homeless website | ***Suggestions for a website dedicated to homeless youth. Include topics, methods of advertisement, title of website, suggestions for a phone/app etc.*** |

| | |
|---|---|
| | I; Why don't you tell me what sort of things would be good on that website?<br>R: Soup kitchens, places for counseling that are free, because a lot of homeless people don't have insurance and stuff like that. |
| Suggestions for Improvement | ***Use this when subject mentions potential improvements/changes that can be made to a website, particular program, anything that can make something they participate in "better".***<br>I:  Any features of them that you wish they had?<br>R:  No.  Yes.  No.  Maybe.  Possibly.  I'm not sure.<br>I:  What are you thinking about?<br>R:  Like if Instagram – I don't know.  I don't know what else you could do to Instagram to make it so much more awesome.  I'm not sure.  Maybe if you could attach Instagram profiles to each other, like closer together. |
| Time | ***Use this node when subject indicates when time is an issue with accessing internet, managing time on the internet, traveling to get to the internet, managing time in general, frequency of use.***<br><br>Ex. R: And I have curfew here and stuff like that, so – and I have things that I have to do during the day that restrict my time on the wifi and my data is gone because I used it all in a day. |
| Uses of the internet | ***Use this node to record specific websites and types of activities a subject visits. This includes using the internet as a source of entertainment, humor, learning it as an educational tool.***<br><br>***Includes examples of what you can access on certain public computers***<br><br>***Includes descriptions of results from web searches***<br>R: I like Vine and Twitter and Facebook and Instagram and I like to play Subway Surfers and I like to listen to music and Don't Tap the White tile and google.  Yeah, that's what I like to do.<br>**You can double-code housing, job searching, or social media as these are separate categories if these are activities people do while on the internet.<br>**If they are explaining specific aspects of the website, such as aspects of a social media site, this should be coded as social media, rather than "uses of the internet". |

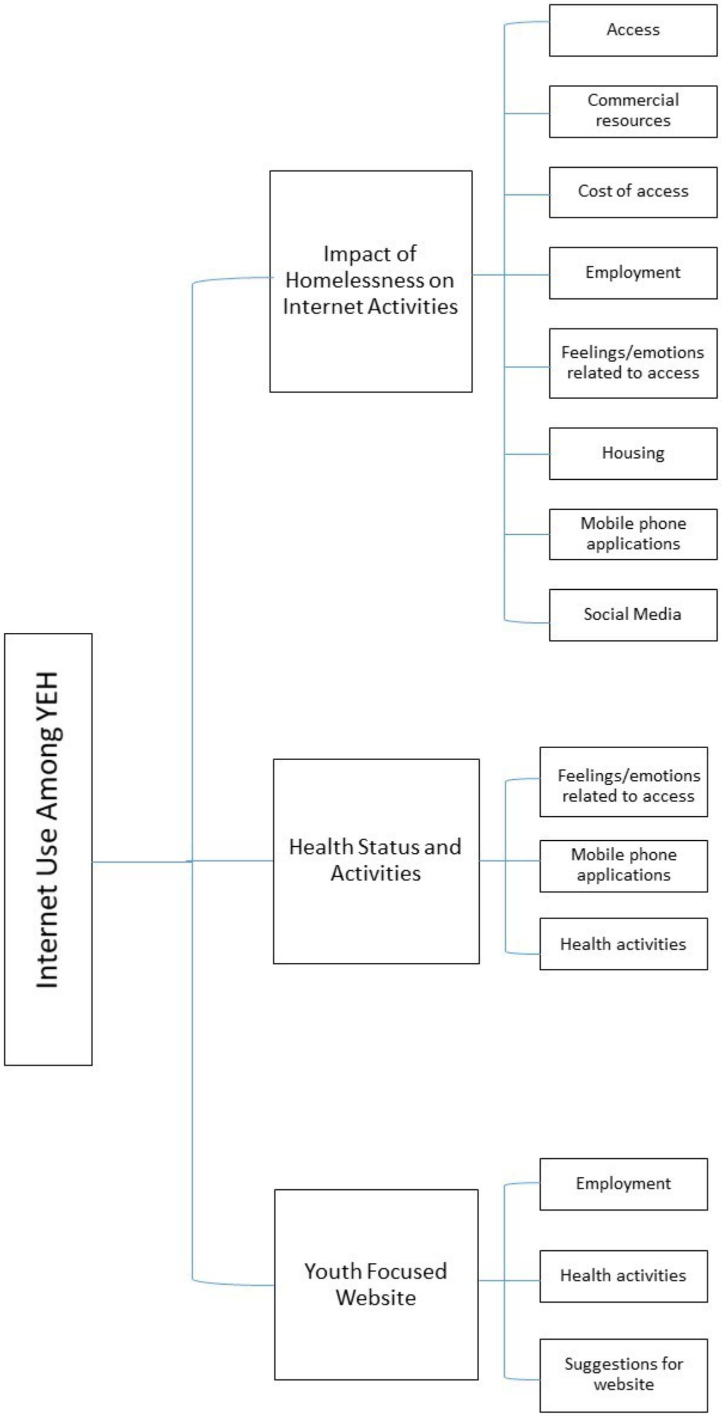
Internet Use Among YEH
Impact of Homelessness on Internet Activities
Access
Commercial resources
Cost of access
Employment
Feelings/emotions related to access
Housing
Mobile phone applications
Social Media
Health Status and Activities
Feelings/emotions related to access
Mobile phone applications
Health activities
Youth Focused Website
Employment
Health activities
Suggestions for website
