# Supplementary material for: Internet and Social Media Access Among Youth Experiencing Homelessness: Mixed-Methods Study
Source: J Med Internet Res. 2018 May 22;20(5):e184. doi: 10.2196/jmir.9306 (PMC5989062; doi:10.2196/jmir.9306)
Supplement: Multimedia Appendix 3 [file jmir_v20i5e184_app3.pdf]

Multimedia Appendix 3: GEE model results

GEE model results for Internet access frequency, time spent using the Internet, social media use, and access to a smartphone regressed on homelessness status, adjusted for age, male gender, and sexual orientation.

Characteristic	Internet access frequency (≥ once/day vs. < once/day)			Time spent using the Internet (more vs. less)			Use of social media (yes vs. no)			Access to a smartphone (yes vs. no)		
	OR	95% CI	*P* value	OR	95% CI	*P* value	OR	95% CI	*P* value	OR	95% CI	*P* value
**Homelessness Status**												
While experiencing homelessness	0.32	0.18-0.57	<.001	0.25	0.16-0.41	<.001	0.13	0.03-0.55	.01	0.33	0.18-0.60	<.001
Before experiencing homelessness	REF	REF		REF	REF		REF	REF		REF	REF	
**Age**												
**Age**	1.32	0.96-1.83	.09	1.10	0.83-1.46	.50	1.21	0.75-1.96	.44	0.88	0.61-1.26	.49
**Male Gender**												
Yes	0.57	0.27-1.17	.13	0.69	0.36-1.31	.26	1.24	0.41-3.76	.71	1.03	0.45-2.37	.94
No	REF	REF		REF	REF		REF	REF		REF	REF	
**Sexual Orientation**												
Heterosexual	0.40	0.10-1.52	.18	2.76	1.04-7.34	.04	1.63	0.44-6.03	.47	0.68	0.20-2.32	.53
Not Heterosexual	REF	REF		REF	REF		REF	REF		REF	REF	

Note: REF=reference category, OR=odds ratio, 95% CI=95% confidence interval
